# Evaluating the Effect of Deficit Irrigation on Yield and Water Use Efficiency of Drip Irrigation Cotton under Film in Xinjiang Based on Meta-Analysis

**DOI:** 10.3390/plants13050640

**Published:** 2024-02-26

**Authors:** Qi Xu, Xiaomei Dong, Weixiong Huang, Zhaoyang Li, Tongtong Huang, Zaijin Song, Yuhui Yang, Jinsai Chen

**Affiliations:** 1 College of Water Conservancy and Architectural Engineering, Tarim University, Alar 843300, China; 10757222200@stumail.taru.edu.cn (Q.X.); 120130023@taru.edu.cn (X.D.); huangwx@cug.edu.cn (W.H.); 120130021@taru.edu.cn (Z.L.); 10757223118@stumail.taru.edu.cn (T.H.); 2 Key Laboratory of Modern Agricultural Engineering, Tarim University, Alar 843300, China; 3 MOE Key Laboratory of Groundwater Quality and Health, School of Environmental Studies, China University of Geosciences, Wuhan 430078, China; 4 Research Office, Beijing City University, Beijing 100193, China; songzaijin@bcu.edu.cn; 5 College of Agronomy and Biotechnology, China Agricultural University, Beijing 100193, China

**Keywords:** drip irrigation under film, meta-analysis, machine learning, cotton yield, water use efficiency, trade-off analysis

## Abstract

Water scarcity constrains the sustainable development of Chinese agriculture, and deficit irrigation as a new irrigation technology can effectively alleviate the problems of water scarcity and water use inefficiency in agriculture. In this study, the drip irrigation cotton field under film in Xinjiang was taken as the research object. Meta-analysis and machine learning were used to quantitatively analyze the effects of different farm management practices, climate, and soil conditions on cotton yield and water use efficiency under deficit irrigation, to investigate the importance of the effects of different factors on cotton yield and water use efficiency, and to formulate appropriate optimization strategies. The results showed that deficit irrigation significantly increased cotton water use efficiency (7.39%) but decreased cotton yield (−15.00%) compared with full irrigation. All three deficit irrigation levels (80~100% FI, 60~80% FI, and 40~60% FI; FI: full irrigation) showed a significant decrease in cotton yield and a significant increase in water use efficiency. Under deficit irrigation, cotton yield reduction was the smallest and cotton water use efficiency increased the most when planted with one film, two tubes, a six-row cropping pattern, an irrigation frequency ≥10 times, a nitrogen application of 300~400 kg·ha^−1^, and a crop density ≥240,000 per hectare, and planted with the Xinluzhong series of cotton varieties; deficit irrigation in areas with average annual temperature >10 °C, annual evapotranspiration >2000 mm, annual precipitation <60 mm, and with loam, sandy soil had the least inhibition of cotton yield and the greatest increase in cotton water use efficiency. The results of the random forest showed that the irrigation amount and nitrogen application had the greatest influence on cotton yield and water use efficiency. Rational irrigation based on optimal management practices under conditions of irrigation not less than 90% FI is expected to achieve a win–win situation for both cotton yield and water use efficiency. The above results can provide the best strategy for deficit irrigation and efficient water use in drip irrigation cotton under film in arid areas.

## 1. Introduction

Cotton is an important cash crop in the world and occupies an important position in world agricultural development [1]. Cotton cultivation in China is mainly distributed in the Yangtze River Basin, the Yellow River Basin, and the northwestern interior. In recent years, with the reduction in cotton planting areas in the Yangtze River and Yellow River basins, Xinjiang has become the largest cotton area in China [2]. The cotton production in Xinjiang accounts for about 90.2% of the total national production [3]. Drip irrigation cotton under film is a combination of drip irrigation technology and film covering technology, which ensures a suitable water and heat environment for cotton growth, reduces the negative impact of soil salinity on plants, promotes seedling growth and development, and significantly improves cotton yield [4,5].

With the successive emergence of problems such as rapid population growth and over-exploitation of groundwater in recent years, China is facing the increasingly severe challenge of water scarcity [6,7]. Water scarcity largely restricts the development of agriculture. According to statistics, China’s agricultural water consumption accounts for about 70% of the total water consumption, with agricultural irrigation accounting for about 90% of the agricultural water use [8,9]. Climate change also constrains the development of water resources, and climate change, mainly global warming, has become one of the most important environmental issues in the world today. Climate change has a direct impact on precipitation, evaporation, soil moisture, etc., making regional water scarcity problems more pronounced [10]. Deficit irrigation is a new type of irrigation technology proposed in response to water scarcity and inefficiency in water use. Deficit irrigation does not pursue the highest yield per unit area and allows for a certain limit of yield reduction. Relevant studies on crops such as alfalfa and wheat have shown that deficit irrigation significantly increases water productivity but reduces crop yields compared to full irrigation [11,12]. Zhang et al. (2016) explored the effects of plant density on cotton growth and yield under deficit irrigation through field experiments, and the results showed that appropriately increasing plant density under deficit irrigation in arid regions is a promising solution to save water without reducing yield [13]; Hou et al. (2021) investigated the response of cotton yield to different irrigation amounts based on a two-year field trial [14]; He et al. (2022) investigated the effects of different irrigation depths on cotton yield and water use efficiency through a field trial [15]; Wang et al. (2018) investigated the effects of different irrigation depths and cotton varieties on cotton yield and water utilization efficiency [16]. In addition, some studies have shown that both soil texture and climatic conditions significantly affect crop yield and water utilization efficiency [17]. Numerous scholars have already conducted multifaceted studies on deficit irrigation, but due to the limitations of experimental conditions and sites, most of the studies have only explored the effects of a small number of factors on cotton yield. Moreover, there are not yet many integrated studies exploring the changing patterns of the yield and water use efficiency of drip irrigation cotton under film. Therefore, a comprehensive analytical method is needed to deeply explore and systematically analyze the existing studies and summarize the effects of deficit irrigation under different conditions, so as to better apply deficit irrigation technology in future practice.

Meta-analysis is the application of statistical concepts and methods to synthesize and quantify the results of a study and to dissect clear patterns of relationships among the variables of interest. Cheng et al. (2021) analyzed the response of cotton yield and water use efficiency to insufficient irrigation under different conditions based on the integration of a global scale but did not systematically study factors such as cotton varieties, planting densities, and cropping patterns [18]. Therefore, this study takes under-film drip irrigation cotton fields as the research object, evaluates the comprehensive effects of deficit irrigation on cotton yield and water use efficiency based on the meta-analysis method, analyzes the importance of the effects of different factors on cotton yield and water use efficiency based on the random forest model, explores the possibility of realizing the mutual balance of the two indexes, and develops suitable optimization schemes through the existing data. The objective of this study is to provide the optimal deficit irrigation strategy and to realize the efficient use of water resources in drip irrigation cotton under film in arid areas.

## 2. Results

### 2.1. Overview of the Database

**Figure 1:**
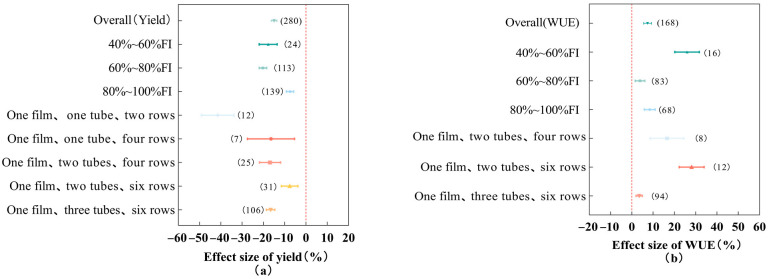
Effects of different irrigation amounts and drip tape modes on cotton yield (**a**) and water use efficiency (**b**). Note: FI is the fully irrigated level. Dots and error lines represent response ratios and their 95% confidence intervals, respectively; non-overlapping of confidence intervals between different subgroups means that the results are significant, and the opposite is not significant. Values in parentheses represent sample sizes, same as below.

The database was derived from 50 literature examples including 280 pairs of observations on cotton yield and 168 pairs of water use efficiency under deficit irrigation conditions, and the frequency distributions of the effect values of cotton yield and water use efficiency conformed to a normal distribution. Compared with full irrigation, deficit irrigation significantly increased cotton water use efficiency by 7.39% (95% *CI*: 5.59~9.18%) but significantly reduced cotton yield (−15.00%, 95% *CI*: −16.36~−13.65%) (Figure 1). The subgroup data can be further analyzed for the specific effects of different farm management practices, soil conditions, and climatic factors on cotton yield and water use efficiency.

### 2.2. Effects of Different Irrigation Amounts and Drip Tape Modes on Cotton Yield and Water Use Efficiency

When the irrigation amount was in the range of 80~100% FI, deficit irrigation reduced cotton yield the least (−7.46%, 95% *CI*: −9.15~−5.77%), which was significantly better than 40~60% FI (−17.74%) and 60~80% FI (−20.21%). As the irrigation amount decreased, the cotton water use efficiency showed a tendency of decreasing and then increasing, as shown in the following: at the irrigation amount of 80~100% FI, the improvement of deficit irrigation on cotton water use efficiency was 8.41% (95% *CI*: 5.86~10.96%); at the irrigation amount of 60%~80% FI, the improvement of deficit irrigation on cotton water use efficiency was 3.83% (95% *CI*: 1.60~6.07%); at the irrigation amount of 40~60% FI, the improvement of deficit irrigation on cotton water use efficiency was 25.95% (95% *CI*: 20.18~31.72%). When drip irrigation tapes were laid in the form of one film and two tubes in six rows, deficit irrigation had the smallest reduction in cotton yield (−7.65%, 95% *CI*: −11.57~−3.73%) and the largest improvement in cotton water use efficiency (28.10%, 95% *CI*: 22.24~33.96%), which was significantly better than one film and two tubes in four rows (16.54%) and one film and three tubes in six rows (3.47%). It can be seen that, under deficit irrigation, both irrigation amount and drip tape mode significantly affected cotton yield and water use efficiency; with the increase in deficit level, both cotton yield and water use efficiency showed the trend of decreasing and then increasing; the drip tape mode of one film, two tubes, and six rows could significantly inhibit the reduction in cotton yield and significantly improve the water use efficiency of cotton.

### 2.3. Effect of Different Irrigation Frequencies and Nitrogen Applications on Cotton Yield and Water Use Efficiency

**Figure 2:**
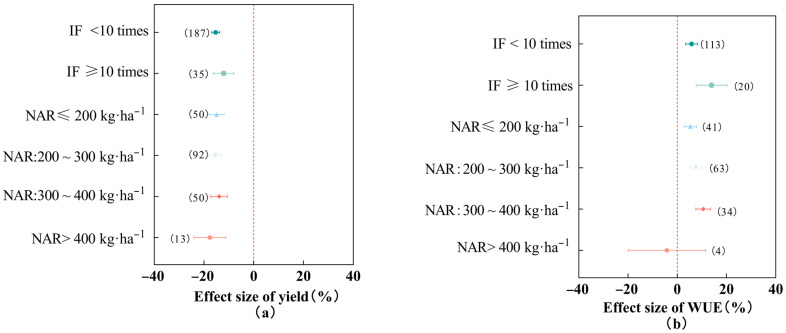
Effects of different irrigation frequencies and nitrogen application rates on cotton yield (**a**) and water use efficiency (**b**). Note: dots and error lines represent response ratios and their 95% confidence intervals, respectively; non-overlapping of confidence intervals between different subgroups means that the results are significant, and the opposite is not significant. Values in parentheses represent sample sizes. IF: irrigation frequency, NAR: nitrogen application rate.

As shown in Figure 2, the reduction in cotton yield by deficit irrigation at irrigation frequencies of ≥10 times (−12.08%, 95% *CI*: −16.21~−7.95%) was lower than the magnitude of reduction in cotton yield at irrigation frequencies of <10 times (−15.29%); and the magnitude of improvement in cotton water use efficiency by deficit irrigation at irrigation frequencies of ≥10 times (13.90%, 95% *CI*: 7.69~20.12%) was higher than the enhancement of cotton water use efficiency at irrigation frequencies of <10 times (5.83%). The reduction in cotton yield with deficit irrigation was lower at nitrogen applications of 300~400 kg·ha^−1^ (−13.91%, 95% *CI*: −17.23~−10.59%) than at ≤200 kg·ha^−1^ (−14.95%), 200~300 kg·ha^−1^ (−15.46%), and >400 kg·ha^−1^ (−17.60%). With the increase in nitrogen applications, the improvement of cotton water use efficiency by deficit irrigation showed a trend of increasing and then decreasing, as shown in the following: under deficit irrigation, cotton water use efficiency was improved by 5.31%, 7.56%, and 10.55% when nitrogen applications were ≤200 kg·ha^−1^, 200~300 kg·ha^−1^, and 300~400 kg·ha^−1^, respectively, but cotton water use efficiency showed an inhibitory effect (−4.19%) when the nitrogen application was >400 kg·ha^−1^. Due to its small sample size (*n* = 4), a larger-scale study was needed to obtain more accurate results. In conclusion, under deficit irrigation, both irrigation frequency and nitrogen application had significant effects on cotton yield and water use efficiency; an irrigation frequency of ≥10 times and a nitrogen application of 300~400 kg·ha^−1^ could effectively inhibit cotton yield reduction and significantly improve cotton water use efficiency.

### 2.4. Effects of Different Varieties and Planting Densities on Cotton Yield and Water Use Efficiency

**Figure 3:**
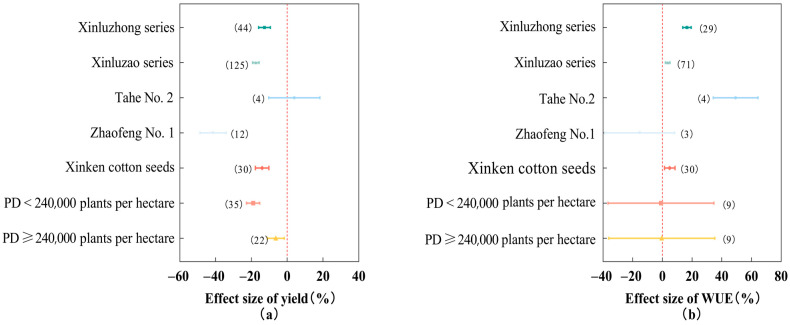
Effects of different varieties and planting density on cotton yield (**a**) and water use efficiency (**b**). Note: dots and error lines represent response ratios and their 95% confidence intervals, respectively; non-overlapping of confidence intervals between different subgroups means that the results are significant, and the opposite is not significant. Values in parentheses represent sample sizes. PD: planting density.

As can be seen in Figure 3. Tahe No. 2 cotton seed showed the advantage of increased yield (3.97%) and efficiency (49.36%) under deficit irrigation; Zhaofeng No. 1 cotton seed showed the disadvantage of reduced yield (−41.47%) and efficiency (−15.23%) under deficit irrigation, and the overall effect was not significant due to the small sample sizes of the two types of cotton seed. Under deficit irrigation, sowing Xinluzhong-series cotton seeds significantly suppressed cotton yield reduction (−12.68%, 95% *CI*: −15.95~−9.41%), which was more effective than Xinluzao-series cotton seeds (−17.52%) and Xinken cotton seeds (−14.00%). Sowing Xinluzhong-series cotton seeds significantly improved cotton water use efficiency (16.55%, 95% *CI*: 13.72~19.39%), which was better than that of Xinluzao-series cotton seeds (3.60%) and Xinken cotton seeds (4.96%). With the increase in cotton planting density, the reduction in cotton yield by deficit irrigation tended to decrease significantly. This was shown as follows: the reduction in cotton yield was 19.04% (95% *CI*: −22.69~−15.38%) at a cotton planting density <240,000 plants per hectare and 6.36% (95% *CI*: −11.09~−1.63%) at a cotton planting density ≥240,000 plants per hectare, whereas the cotton water use efficiency did not differ significantly among the planting densities of different levels. In conclusion, the Xinluzhong series of cotton varieties could significantly inhibit the cotton yield reduction and significantly increase the cotton water utilization efficiency under deficit irrigation conditions, and the crop density of ≥240,000 plants per hectare could significantly inhibit the cotton yield reduction.

### 2.5. Effects of Different Climatic and Soil Conditions on Cotton Yield and Water Use Efficiency

**Figure 4:**
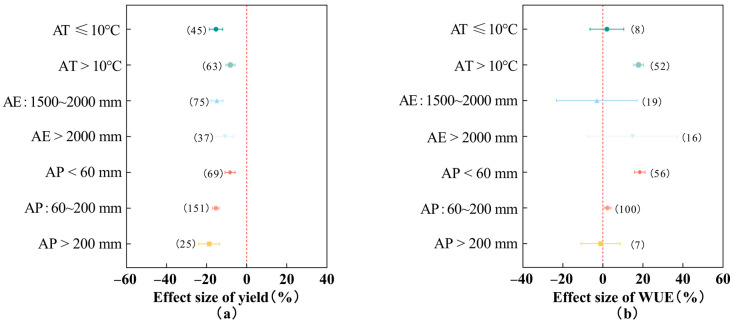
Effects of different climatic conditions on cotton yield (**a**) and water use efficiency (**b**). Note: dots and error lines represent response ratios and their 95% confidence intervals, respectively; non-overlapping of confidence intervals between different subgroups means that the results are significant, and the opposite is not significant. Values in parentheses represent sample sizes. AT: annual temperature, AE: annual evaporation, AP: annual precipitation.

As shown in Figure 4, the reduction in cotton yield due to deficit irrigation in areas with a mean annual temperature >10 °C (−8.15%, 95% *CI*: −10.45~−5.85%) was significantly lower than that in areas with a mean annual temperature ≤10 °C (−15.31%), and the enhancement of cotton water use efficiency due to deficit irrigation (17.80%, 95% *CI*: 15.24~20.36%) was significantly higher (1.99%) than in areas with a mean annual temperature ≤10 °C. With the increase in annual evaporation, the reduction in cotton yield by deficit irrigation gradually decreased and the improvement of cotton water use efficiency gradually increased, as shown in the following: under deficit irrigation, the reduction in cotton yield at an annual evaporation of 1500~2000 mm was 14.90% (95% *CI*: −17.90~−11.89%), and the reduction in cotton yield at an annual evaporation >2000 mm was 10.88% (95% *CI*: −15.35~−6.42%). The increases in cotton water use efficiency were −3.03% for an annual evaporation of 1500~2000 mm (which showed an inhibitory effect) and 14.84% for an annual evaporation of >2000 mm. With the increase in annual precipitation, the decrease in deficit irrigation on cotton yield gradually increased, and the increase in cotton water use efficiency gradually decreased, as shown in the following: under deficit irrigation conditions and annual precipitation amounts of <60 mm, 60~200 mm, and >200 mm, cotton yield decreased by 8.32%, 15.42%, and 18.72%, respectively; cotton water use efficiency increased by 18.43%, 2.27%, and −1.14%, respectively (at an annual precipitation >200 mm, the cotton water use efficiency showed an inhibitory effect). Therefore, deficit irrigation in areas with average annual temperature >10 °C, annual evaporation >2000 mm, and annual precipitation <60 mm had the least inhibitory effect on cotton yield and the greatest increase in cotton water use efficiency.

**Figure 5:**
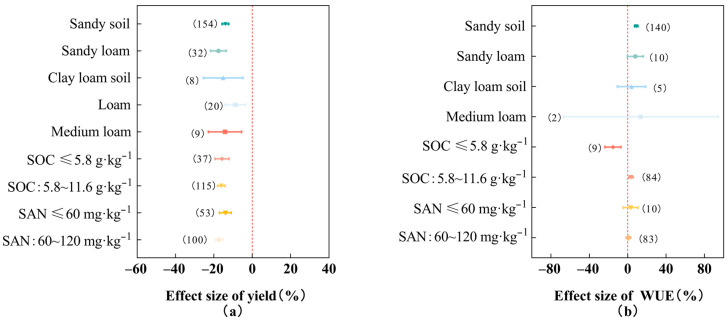
Effects of different soil conditions on cotton yield (**a**) and water use efficiency (**b**). Note: dots and error lines represent response ratios and their 95% confidence intervals, respectively; non-overlapping of confidence intervals between different subgroups means that the results are significant, and the opposite is not significant. Values in parentheses represent sample sizes. SOC: soil organic carbon, SAN: soil available nitrogen.

As shown in Figure 5, deficit irrigation had the least reduction in cotton yield when the soil was of a loam texture (−8.73%, 95% *CI*: −14.02~−3.44%), followed by a sandy soil texture (−13.98%), while the greatest reduction in cotton yield was observed when the soil was of a sandy loam texture (−17.63%). The greatest improvement in cotton water use efficiency was observed in soil with a medium loam texture (13.51%, 95% *CI*: −67.26~94.28%), but the overall boost was not significant due to the small sample size (*n* = 2) and the 95% confidence interval containing zero. Deficit irrigation in soil with a sandy soil texture significantly improved cotton water use efficiency (8.83%, 95% *CI*: 7.03~10.63%), and the enhancement was better than that in a sandy loam soil texture (7.93%) and clay loam soil texture (3.94%). Cotton yield did not differ significantly among the different levels of initial soil organic carbon, but the cotton field soil initial organic carbon content of 5.8~11.6 g·kg^−1^ significantly improved cotton water use efficiency (3.41%, 95% *CI*: 1.13~5.70%). Deficit irrigation at a soil available nitrogen content ≤60 mg·kg^−1^ in a cotton field could effectively inhibit cotton yield reduction (−14%), and the effect was better than that of a soil available nitrogen content of 60~120 mg·kg^−1^ (−17.37%); the increase in cotton water use efficiency at a soil available nitrogen content ≤60 mg·kg^−1^ in a cotton field (3.11%) was greater than that of a soil available nitrogen content of 60~120 mg·kg^−1^ (0.69%), but there was no significant difference between the two levels. In conclusion, deficit irrigation in loam and sandy soils showed the least reduction in cotton yield, and deficit irrigation in sandy soils showed the greatest increase in cotton water use efficiency.

### 2.6. Trade-Offs between Cotton Yield and Water Use Efficiency under Deficient Irrigation and Their Key Influencing Factors

**Figure 6:**
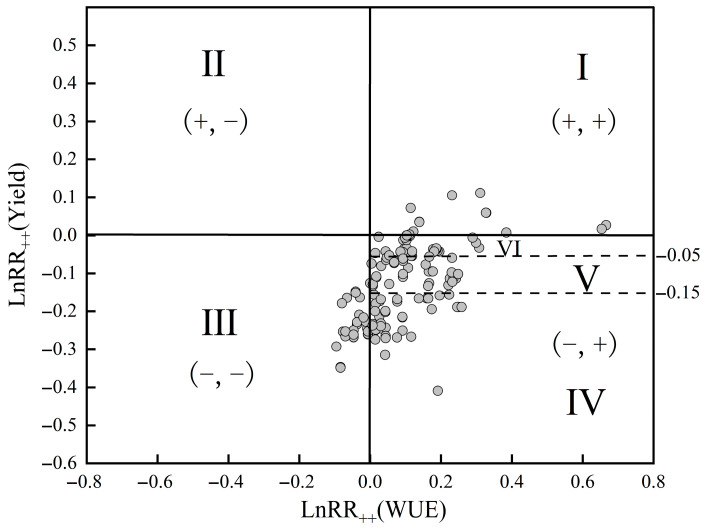
Effect distribution of cotton yield and water use efficiency on deficit irrigation response based on all paired data in regions I, II, III, IV, and V. Note: the area with 5% cotton yield reduction and water use efficiency greater than 0 is divided into VI. The data points in the figure are the matching data points of cotton yield and water use efficiency, and the horizontal line in the figure represents the critical line of 5% cotton yield reduction and 15% cotton yield reduction.

In order to better understand the equilibrium relationship between deficit irrigation on cotton yield and water use efficiency, in this study, 168 data points of cotton yield and water use efficiency were plotted in the plane of Figure 6. All the paired effect values of yield and water use efficiency were classified into four regions, where region I represents the increase in both cotton yield and water use efficiency, region II represents the increase in cotton yield and water use efficiency decrease, region III represents the decrease in both cotton yield and water use efficiency, region IV represents the increase in water use efficiency with a decrease in cotton yield, and region V represents the realization of an increase in cotton water use efficiency under the condition that the cotton yield is reduced by less than 15.00%. Among which, region I represents a win–win situation, although region I is the expected goal of practice, but with only 14 pairs of data, accounting for the total number of strongholds of 8.44%. The low percentage of data volume also indicates that it is difficult to realize the mutual improvement of cotton yield and water use efficiency by deficit irrigation under the existing management measures, and appropriate irrigation strategies should be adopted to realize the trade-off between cotton yield and water use efficiency. The absence of data points in region II also showed that there was no increase in cotton yield but a decrease in water use efficiency under deficit irrigation. Data points in regions III, IV, and V accounted for 19.27%, 72.29%, and 45.78% of the total, respectively. Although it is difficult to achieve a win–win situation for both cotton yield and water use efficiency, the 45.78% data share within region V indicates that there was a slight decrease in cotton yield and an increase in water use efficiency in most of the studies.

**Figure 7:**
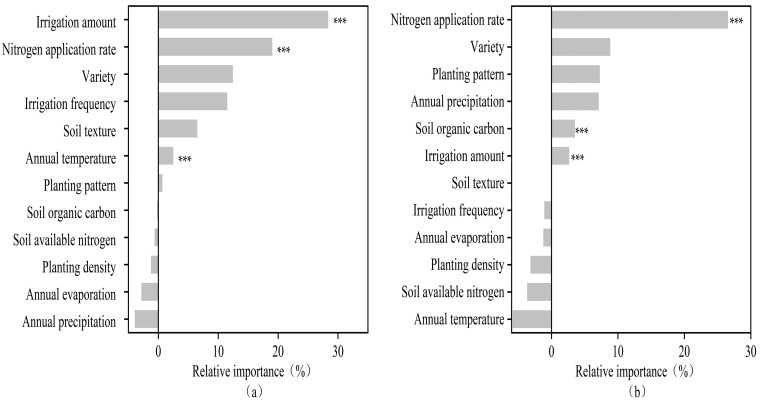
The relative importance of each factor to cotton yield (**a**) and water use efficiency (**b**) under the random forest model, with the *** sign in the figure representing the significance level.

Importance analysis of the factors affecting cotton yield and water use efficiency was carried out using random forest, and the results are shown in Figure 7. Relative importance represents the degree of influence of each factor on cotton yield and water use efficiency, and the larger the value, the greater the importance of the influence of the variable [19]. The * sign in the figure represents the significance level of each variable. As shown in the figure, the factors that had a greater effect on cotton yield and reached a highly significant level were irrigation amount, nitrogen application, and average annual temperature. The factors that had a significant effect on cotton water use efficiency at the highly significant level were nitrogen application, initial soil organic carbon content, and irrigation amount.

In this study, the area with a 5% cotton yield reduction and water use efficiency >0 was divided into region VI, which represents the lowest cotton yield reduction acceptable to this study. Regions I and VI were the target regions, and this study analyzed the data from different groups of irrigation amount, nitrogen application, and average annual temperature, which had significant effects on cotton yield, in the target and other regions (Table 1). Among them, the proportion of data points in the target region was much larger than that in other regions for a nitrogen application of 300~400 kg·ha^−1^ and a nitrogen application of >400 kg·ha^−1^, while the other groups showed that the proportion of data in the target region was smaller than that in other regions, or the proportion of data in the target region was similar to that in other regions.

In order to construct a suitable deficit irrigation strategy to realize the rational use of agricultural water resources and promote the win–win situation of cotton yield and water use efficiency in the target area, this study conducted subgroup analysis of irrigation water quantity in the target area. The results, as shown in Table 2, showed that the mean effect size of deficit irrigation on cotton yield in the target area was −0.99%, and the negative effect of deficit irrigation on cotton yield gradually decreased with the increase in irrigation amount, and the decrease in deficit irrigation on cotton yield was the smallest (−0.16%) and was significantly larger than that of −0.99% when the irrigation amount was 90%~100% FI. In other words, rational irrigation according to optimal management measures under an irrigation amount of not less than 90% FI is expected to achieve a win–win situation of cotton yield and water use efficiency.

## 3. Discussion

### 3.1. The Combined Effect of Deficient Irrigation on Cotton Yield and Water Use Efficiency

In this study, the combined effect of deficit irrigation on cotton yield and water use efficiency was quantified based on the meta-analysis method by using the valid data of 280 pairs of cotton yield and 168 pairs of cotton water use efficiency from 50 literature examples. The results of the study showed that deficit irrigation increased cotton water use efficiency (7.39%) but significantly reduced cotton yield (−15.00%) compared to full irrigation. Different management practices (irrigation amount, drip tape mode, etc.), climate, and soil conditions significantly affected cotton yield and water use efficiency. Therefore, it is necessary to analyze the effects of different subgroups on cotton yield and water use efficiency and develop suitable optimization strategies under deficit irrigation to minimize the reduction in cotton yield and significantly improve cotton water use efficiency.

### 3.2. Effects of Different Farm Management Practices on Cotton Yield and Water Use Efficiency

The research content of this experiment is about drip irrigation cotton under film, which is a combination of drip irrigation technology and film covering technology [20], to ensure the appropriate water and heat environment for cotton growth, so that the cotton fertility period can be advanced and cotton yield can be significantly increased [4]. The results of this study showed that the drip tape mode of one film, two tubes, and six rows could significantly inhibit cotton yield reduction and significantly improve cotton water utilization efficiency. The planting pattern of one film, two tubes, and six rows has a good thermal insulation effect, and the appropriate soil temperature is favorable to the early growth and development of cotton [21]. Some studies have shown that the drip tape mode of double tubes is better than single tubes in terms of the salt control effect [22,23]. Too much soil salt will inhibit the absorption of nutrients by the cotton root system, thus affecting the normal growth and development of cotton; too much salt leads to a decrease in the infiltration type of soil water, which significantly reduces the soil water content, leading to the shortage of water caused by the drought stress of cotton. Therefore, the planting pattern of one film, two tubes, and six rows under deficit irrigation can create a desalinated soil environment for the cotton root zone, significantly improve the water utilization efficiency of cotton, and inhibit the reduction in cotton yield. This study showed that deficit irrigation reduced cotton yield by 15% and increased water use efficiency by 7.39%. An irrigation ration of 80~100% FI had the smallest reduction in cotton yield (7.46%), and the appropriate range of moisture changes did not significantly reduce cotton yield [24]; this study found that a 20% to 25% water deficit had less effect on cotton yield [25,26], which may be due to the fact that drip irrigation under film reduces the ground evaporation between plants and allows the soil to remain loose and water-laden [27]. When the irrigation amount of 80~100% FI decreased to 60~80% FI, the cotton yield decreased significantly, but the water use efficiency did not increase significantly. It has been suggested that an increase in water use efficiency under limited water conditions does not equate to high yields, even if yields are significantly reduced, which is mainly dependent on the transpiration and photosynthetic capacity of leaves in response to water stress [11,28].

Under deficit irrigation, with the increase in irrigation frequency, the yield reduction of cotton gradually decreases, and the improvement of water use efficiency gradually increases. Appropriately increasing the frequency of irrigation can effectively reduce the risk of cotton yield reduction. Studies by some scholars have shown that an increase in the frequency of irrigation can increase crop yield [29,30]. High-frequency irrigation concentrates water in a small area of the root zone, which promotes the growth of the cotton root system and the uptake and utilization of nutrients. Previous studies have shown that the appropriate amount of nitrogen application can increase cotton yield and water utilization efficiency [18]. A study of the coupling of water and nitrogen conducted by Zhong Deng et al. (2015) in south Xinjiang showed that a nitrogen application of 300 kg·ha^−1^ promoted cotton yield at different irrigation water levels [31]. Appropriate fertilizer application contributes to crop growth and development and photosynthesis [32]; it helps to improve crop water use efficiency [33]. Excessive nitrogen application leads to a plant nutrient imbalance detrimental to cotton growth and yield formation and aggravates nitrogen loss and environmental burdens [34]. In this study, we showed that under deficit irrigation, an irrigation frequency ≥10 times and a nitrogen application of 300~400 kg·ha^−1^ increased cotton water use efficiency the most and significantly suppressed cotton yield reduction.

The overall effect of the Tahe No. 2 and Zhaofeng No. 1 cotton varieties was not significant due to the small sample size, and a larger study is needed to obtain more accurate results. Therefore, this study focuses on the effects of Xinluzhong-series cotton varieties, Xinluzao-series cotton varieties, and Xinken cotton 1 on cotton yield and water use efficiency. As can be seen from Figure 4, Under deficit irrigation conditions, Xinluzhong-series cotton varieties had the smallest reduction in cotton yield and the largest increase in water use efficiency compared with the other two types of cotton varieties. Xinluzhong-series and Xinluzao-series cotton varieties belong to the same Xinjiang cotton varieties. Xinluzhong-series cotton varieties are medium-maturity land cotton varieties, which are generally stronger in disease resistance and yield than the Xinluzao-series cotton varieties. Xinken cotton 1 and Xinluzao-series cotton varieties belong to the same category of early maturity land cotton, but they are more suitable to be planted in the Hexi Corridor cotton area [35]. As far as crop planting density is concerned, it has been shown that under deficit irrigation, high planting density can conserve water without decreasing seed cotton yield [13,18,36]. Chen’s study showed that limited irrigation conditions and high planting density can obtain larger crop yields [37], which are all consistent with the results of this study. The results of this study showed that a crop planting density of ≥240,000 plants per hectare could significantly inhibit cotton yield reduction.

### 3.3. Effect of Different Climatic and Soil Conditions on Cotton Yield and Water Use Efficiency

The response of cotton yield and water use efficiency to deficit irrigation was significantly influenced by climate and soil conditions. The results of this study showed that deficit irrigation in areas with an average annual temperature >10 °C, an annual evaporation >2000 mm, and an annual precipitation <60 mm had the least inhibition of cotton yield and the greatest increase in cotton water use efficiency. Cotton is a thermophilic crop, and a higher-temperature environment is favorable for cotton growth and water use; low temperature will prolong the cotton fertility period and, thus, lead to a decrease in cotton yield [38]. Some studies have shown that a high evaporation rate reduces plant moisture and significantly reduces boll yield [39], which is inconsistent with the results of the present study, probably because the study area is an arid zone with low precipitation and high evaporation, and the cotton species is highly drought-tolerant. The reduction in precipitation leads to a decrease in evapotranspiration of the cotton species, which in turn increases the water use efficiency. As far as soil texture is concerned, loam and sandy soils under deficit irrigation conditions showed the least reduction in cotton yield, and sandy soils showed the greatest increase in cotton water use efficiency. Sandy soil has the advantages of rough particles and good permeability. Some scholars’ studies have concluded that rough-textured soil has higher hydraulic conductivity, which is more favorable to plant root growth [12]; loam soil combines the advantages of both clay and sandy soil [40,41], has strong resistance to stress and drought and flooding, and can inhibit the reduction in cotton yields under deficit irrigation conditions. In terms of soil initial organic carbon and soil available nitrogen content, there is no significant effect on cotton yield and water use efficiency among different subgroups under deficit irrigation conditions. A too-high initial soil fertility will lead to vigorous microbial activity in the soil, which will consume soil oxygen and reduce cotton yield; a too-low initial soil fertility will also inhibit cotton growth. In addition, the drip irrigation cotton under film was supplied with fertilizer by drip application with water through the fertilizer tank, and the drip application of fertilizers with water ensured that the water and fertilizer solutions were uniformly distributed in the soil, which allowed the nutrients to be fully utilized. This may also be the reason why the initial soil fertility did not have a significant effect on cotton yield and water use efficiency.

## 4. Materials and Methods

### 4.1. Data Collection

The literature data adopted in this study were obtained from the China National Knowledge Infrastructure (www.cnki.net) and Web of Science (www.webofscience.com). The keywords of the literature search included cotton, drip irrigation under film, drip irrigation, cotton yield, and yield. Excluding the water quality of irrigation, such as brackish water, magnetized water, etc., we screened scholars’ published journal papers and master’s doctoral dissertations from 1 January 1960 to 31 December 2022. According to the needs of the research topic, the literature screening for inclusion needed to meet the following criteria: (1) the test site should be a field trial within the scope of Xinjiang, potting and indoor trials should be excluded, and the test object should be cotton; (2) the number of trial replicates should be ≥3; (3) cotton yields recorded in the study cases were seed cotton yields; (4) multiple replicated observations under the same treatment were averaged; (5) when there were studies with additional factor effects in the trials, they were considered as independent trials separately included in the database; (6) for multi-year experimental studies in the same region in the literature, they were considered to be mutually independent trials separately included in the database. In addition, based on scholars’ studies on deficit irrigation in Xinjiang [37,42,43], this study defined the range of complete irrigation (full irrigation) as between 368 and 425 mm, and the irrigation amount below this range was considered as deficit irrigation and classified into three levels (40~60% FI, 60~80% FI, and 80~100% FI, FI being the fully irrigated level). The irrigation amount described in this study is the sum of successive irrigation volumes over the entire cotton reproductive period, which consists of four periods: seedling, bud, boll blossom, and maturity. The data in the literature tables were extracted directly, and for the data in the bar charts and line graphs, the software Get Data 2.20 was used to digitize the images before extraction [44]. According to the above criteria, 50 documents and 448 sets of valid data were finally screened and obtained in this study. The names of the literature included in this study and the distribution of the test sample sites were documented in the additional material.

### 4.2. Meta-Analysis

In order to ensure the reliability of the study, the weighted response ratio (*RR*) was used as a statistical indicator in this study. The natural logarithm of the weighted response ratio (*LnRR*) was used to express the degree of influence of a particular driving factor, and its 95% confidence interval (95% *CI*) was calculated. Its calculation formula is [45]:

(1) RR=Xe/Xc

Taking cotton yield as an example, Xe is the average yield of cotton under deficit irrigation (kg·ha^−1^); Xc is the average yield of cotton under full irrigation (kg·ha^−1^).

(2) LnRR=Ln(Xe/Xc)

If *LnRR* > 0, it means that deficit irrigation has a positive effect on cotton yield, which shows the promotion effect; if *LnRR* < 0, it means that deficit irrigation has a negative effect on cotton yield, which shows the inhibition effect, and the larger the value of *LnRR* is, the more obvious the effect is [46].

The response ratios of each study were weighted together to produce an average weighted response ration (*RR*++). In addition, variance (*V*), weighted factor (*W*), *RR*++ standard deviation, and 95% confidence intervals were obtained by calculating Equations (3)–(7).

(3) $$RR_{++}=\frac{\sum_{i=1}^{m}\sum_{j=1}^{ki}W_{ij}RR_{ij}}{\sum_{i=1}^{m}\sum_{j=1}^{ki}W_{ij}}$$

where *m* is the number of subgroups, e.g., different varieties or soil texture subgroups, and *ki* represents the number of pairs of data in the *i*th subgroup.

(4) $$V=\frac{SDe^{2}}{NeXe^{2}}+\frac{SDc^{2}}{NcXc^{2}}$$

*SDe*^2^ and *SDc*^2^ represent the standard deviation of the test and control groups, respectively. For missing *SD* values in the literature, 1/10 of the mean of the corresponding test or control group was used as a substitute; *Ne* and *Nc* represent the number of samples in the test and control groups [47].

(5) $$W=\frac{1}{V}$$

(6) $$S(RR_{++})=\sqrt{\frac{1}{\sum_{i=1}^{m}\sum_{j=1}^{ki}W_{ij}}}$$

(7) $$95\%CI=RR_{++}\pm 1.96S(RR_{++})$$

To better understand the results of the meta-analysis, 95% confidence intervals were transformed into percent change by (*e*^(*LnRR++)*−1)^ × 100%). When the 95% *CI* was far from the zero line, it indicated that the treatment group was significantly different from the control group (*p* < 0.05); when the 95% *CI* intersected with the zero line, it indicated that there was no significant difference between the treatment group and the control group (*p* > 0.05).

### 4.3. Publication Bias Test

**Figure 8:**
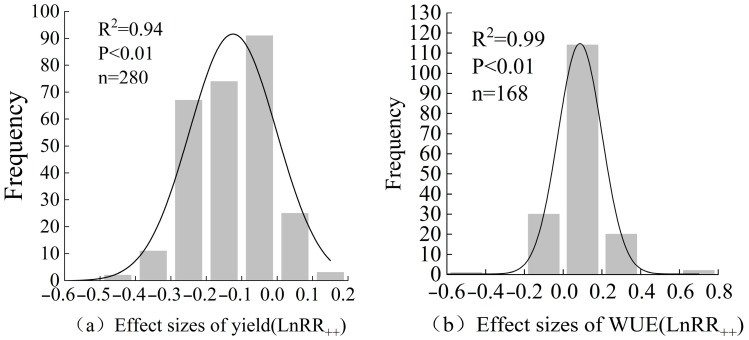
Frequency distribution of the effects of deficit irrigation on cotton yield (**a**) and water use efficiency (**b**). *n* is the number of sample sizes, and *p* is the significance level.

The chi-square test (*CST*) was performed on the data to clarify whether there was clear heterogeneity among treatments. According to the results, the corresponding fixed-effect model or random-effect model was adopted; if *p* > 0.05, it indicated that there was no significant difference in the results of the studies, and the fixed-effect model was adopted for the calculations. On the contrary, the random-effect model was adopted for the calculations. The bias test was conducted by the fail-safe number (*Nfs*) method; if *Nfs* > 5*n* + 10 (*n* is the logarithm of data), it means that there is no publication bias, and the conclusion is credible [11]. The significance tests of cotton yield and water use efficiency in this study have *p* values < 0.001, which indicates that a random-effects model should be adopted to carry out subgroup analyses to explain the heterogeneity in depth; the *Nfs* values of cotton yield and water use efficiency are 48,172.8 and 3945.7, respectively. The numbers of pairs of data are 280 and 168 pairs, the *Nfs* is much larger than 5*n* + 10, and the cotton yield and water use efficiency effect values frequencies conformed to normal distribution (Figure 8), which indicated that there was no publication bias in this study and that the conclusions were reliable.

### 4.4. Subgroup Analysis

Different farm management practices (irrigation amount, planting mode, variety, etc.) have a large impact on cotton yield and water use efficiency, while many factors such as climate and soil conditions also have an effect on them. Therefore, in this study, the literature data were grouped in multiple ways for irrigation amount [11], drip tape mode, irrigation frequency [38], nitrogen application [18], variety, planting density [37], average annual temperature [12], annual evapotranspiration [48], annual precipitation [49,50], soil texture, and initial soil fertility [51,52] (Table 3).

### 4.5. Statistical Analysis

Microsoft Office 2016 software (Microsoft, Redmond, WA, USA) was used to complete the database and some of the data calculations; MetaWin 2.1 (State University of New York at Stony Brook, New York, NY, USA) was used to complete the meta-analysis of the random-effects model; GraphPad Prism 8 (GraphPad Software, Boston, MA, USA) and Origin 2021 (OriginLab, Northampton, MA, USA) were used to complete the graphs; and R 4.3.2 statistical software (The University of Auckland, Auckland, New Zealand) was used to perform the random forest importance analysis.

## 5. Conclusions

In this study, the combined effects of deficit irrigation on cotton yield and water use efficiency were analyzed in depth. The results showed that deficit irrigation reduced cotton yield by 15.00% and increased water use efficiency by 7.39% compared with full irrigation. The specific effects of different farm management practices and climatic conditions on cotton yield and water use efficiency were further analyzed by subgroup data. The results showed that all three deficit irrigation levels showed significant decreases in cotton yield and significant increases in water use efficiency. Under deficit irrigation, the conditions of a drip tape mode with one film, two tubes, and six rows, an irrigation frequency of ≥10 times, and a nitrogen application of 300~400 kg·ha^−1^ could effectively suppress the cotton yield reduction and significantly improve the cotton water use efficiency; the sowing of the Xinluzhong series of cotton seeds and a crop density of ≥240,000 plants per hectare effectively inhibited the reduction in cotton yield and significantly improved the cotton water use efficiency. In the area with an average annual temperature >10 °C, an annual evaporation >2000 mm, an annual precipitation <60 mm, and loam and sandy soil, deficit irrigation had the smallest inhibition of cotton yield and the largest increase in cotton water use efficiency. Among them, irrigation amount and nitrogen application had the greatest effect on cotton yield and water use efficiency. Rational irrigation based on optimal management practices under conditions of irrigation not less than 90% FI is expected to achieve a win–win situation for both cotton yield and water use efficiency.

## Figures and Tables

**Table 1:** Proportions of different subgroups of irrigation amount, nitrogen application rate, and annual mean temperature in the target area and other regions.

Impact Factor	Category	Target Zone	Other Zone
Irrigation amount, %	40~60% FI	13.33%	86.67%
	60~80% FI	23.73%	76.27%
	80~100% FI	55.74%	44.26%
Nitrogen application rate, kg·ha^−1^	≤200	21.21%	78.79%
	200~300	31.25%	68.75%
	300~400	62.07%	37.93%
	>400	100%	0%
Average annual temperature, °C	≤10	50.00%	50.00%
	>10	58.00%	42.00%

**Table 2:** Analysis of optimized irrigation volumes within the target region.

Impact Factor	Category	Effect Value of Cotton Yield	95% Confidence Interval	Data Sample Size	Mean Effect Size
Irrigation amount, %	80~90% FI	−1.23%	−3.53~1.07%	25	−0.99%
	90~100% FI	−0.16%	−4.86~4.53%	11	

**Table 3:** Data grouping.

Categorical Variables	Groups
Irrigation amount, %	40~60% FI; 60~80% FI; 80~100% FI
Drip tape mode	one film, one tube, two rows; one film, one tube, four rows; one film, two tubes, four rows; one film, two tubes, six rows; one film, three tubes, six rows
Irrigation frequency, times	<10; ≥10
Nitrogen application rate, kg·ha^−1^	≤200; 200~300; 300~400; >400
Variety	Xinluzhong series; Xinluzao series; Tahe No. 1; Zhao feng No. 2; Xinken cotton seeds
Planting density, plants·ha^−1^	<240,000; ≥240,000
Annual temperature, °C	≤10; >10
Annual evaporation, mm	1500~2000; >2000
Annual precipitation, mm	<60; 60~200; >200
Soil texture	Sandy soil; Sandy loam; Loam; Clay loam soil; Medium loam
Soil organic carbon, g·kg^−1^	≤5.8; 5.8~11.6
Soil available nitrogen, mg·kg^−1^	≤60; 60~120

## Data Availability

The data already exist in the manuscript and Supplementary Materials.

## Supplementary Materials

The following supporting information can be downloaded at https://www.mdpi.com/article/10.3390/plants13050640/s1. Section S1. Classification of variables; Figure S1. Literature Screening Flowchart; Table S1. Description of categorical variables; Table S2. Sample size and significance test for categorical variables; Table S3. Distribution of titles and locations of the literature included in the database.
