# Supplementary material for: Evaluating the Effect of Deficit Irrigation on Yield and Water Use Efficiency of Drip Irrigation Cotton under Film in Xinjiang Based on Meta-Analysis
Source: Plants (Basel). 2024 Feb 26;13(5):640. doi: 10.3390/plants13050640 (PMC10935209; doi:10.3390/plants13050640)
Supplement: Supplementary file 1 [file plants-13-00640-s001.zip › Supplementary material.pdf]

Supplementary material

Evaluating the effect of deficit irrigation on yield and water use efficiency of drip irrigation cotton under film in Xinjiang based on Meta-analysis

Qi Xu[1,2†], Xiaomei Dong[1†], Weixiong Huang[1,3], Zhaoyang Li[1,2], Tongtong Huang[1], Zaijin Song[4], Yuhui Yang[1,2*] and Jinsai Chen[5*,]

1 College of Water Conservancy and Architectural Engineering, Tarim University, Alar 843300, China; 10757222200@stumail.taru.edu.cn (Q.X.); 120130023@taru.edu.cn (X.D.); huangwx@cug.edu.cn (W.H.); 120130021@taru.edu.cn (Z.L.); 10757223118@stumail.taru.edu.cn (T.H.)

2 Key Laboratory of Modern Agricultural Engineering, Tarim University, Alar 843300, China

3 MOE Key Laboratory of Groundwater Quality and Health, School of Environmental Studies, China University of Geosciences, Wuhan 430078, China

4 Beijing City University, Beijing 100193, China; songzaijin@bcu.edu.cn

5 College of Agronomy and Biotechnology, China Agricultural University, Beijing 100193, China

* Correspondence: 120130022@taru.edu.cn (Y.Y.); jinsaichen@cau.edu.cn (J.C.)

† These authors contributed equally to this work.

S1 Classification of variables

Fig. S1 Literature Screening Flowchart

Table S1 Description of categorical variables

Table S2 Sample size and significance test for categorical variables

Table S3 Distribution of titles and locations of literature included in the database

S1 Classification of variables

In this study, twelve variables were evaluated based on the availability of the dataset and combined with previous categorizations. Irrigation amount (40~60% FI, 60~80% FI, 80~100% FI), irrigation frequency (<10, ≥10), nitrogen application (≤200, 200~300, 300~400, >400), planting density (<240000, ≥240000), average annual temperature (≤10, >10), average annual precipitation (<60, 60~200, > 200), average annual evapotranspiration (1500~2000, >2000), initial soil organic carbon content (≤5.8, 5.8~11.6), and initial soil available nitrogen content (≤60, 60~120), of which the drip tape mode, variety and soil texture were categorized according to the different categories in the available database. Soil organic matter was categorized as ≤5, 5~10, 10~15, 15~20, >20 g/kg. In this study, soil organic carbon was analyzed, and according to the "Van Bemmelen" factor, soil organic carbon=soil organic matter*0.58, and according to references, soil organic matter was categorized as ≤10, 10~20, which was converted to ≤5.8, 5.8~11.6.

Reference: Bian, Q.Q.; Wang, Y.N.; Chen, J.J.; Qiao, S.C.; Hu, L.L.; Yin, Y.M.; Yang, X. P.; Yang, Y.F. Effects of potassium application on yield and potassium balance of sweet potato field in China: A meta-analysis. J. Plant Nutr. Fertil. 2022, 28, 1509–1519. https://doi.org/10.11674/zwyf.2021632.（Reference is cited in the text）

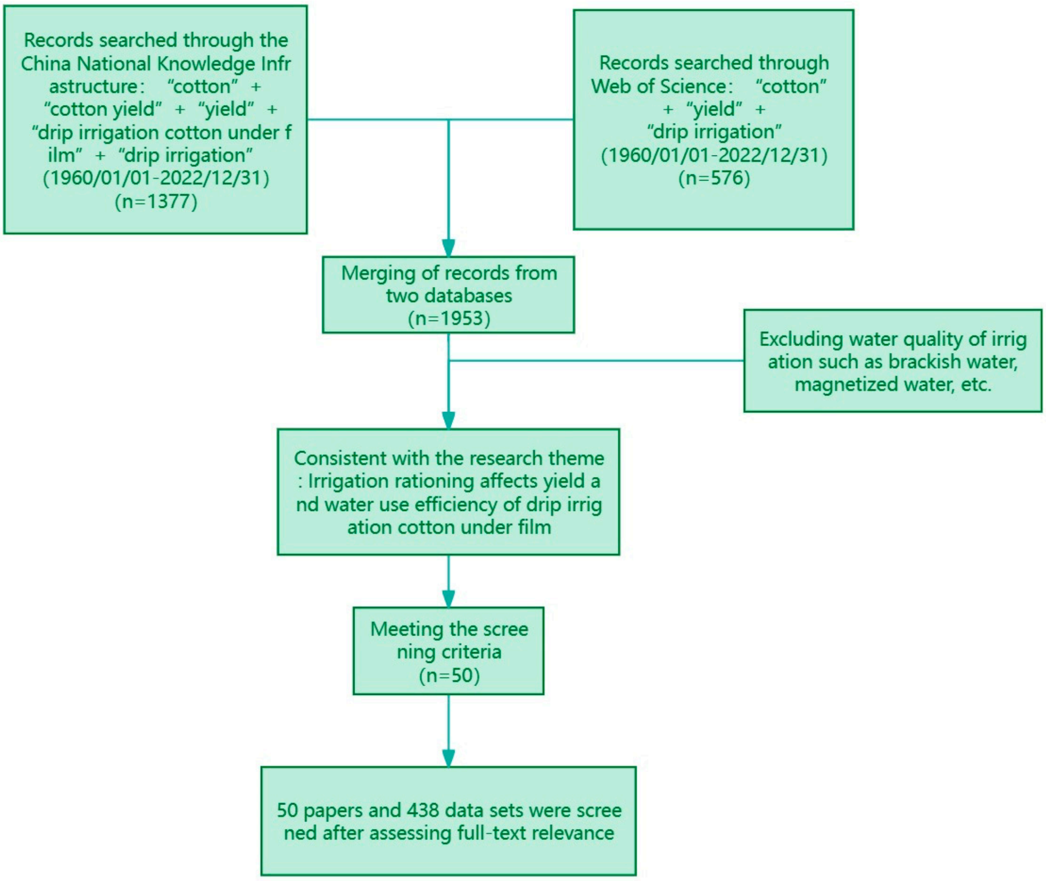

Figure S1 Literature Screening Flowchart

Figure S1 shows the flowchart of screening literature for this study. The screening criteria followed in this study were (1) the test site should be a field trial within the scope of Xinjiang, potting and indoor trials should be excluded, and the test object should be cotton; (2) the number of trial replicates should be ⩾3; (3) cotton yields recorded in the study cases were seed cotton yields; (4) multiple replicated observations under the same treatment were averaged; (5) when there were studies with additional factor effects in the trials, they were considered as independent trials separately included in the database; (6) for multi-year experimental studies in the same region in the literature, they were considered to be mutually independent trials separately included in the database.

Table S1 Description of categorical variables

Category	Variable	Unit
Management measures	Irrigation amount	%
	Irrigation frequency：IF	times
	Nitrogen application rate：NAR	kg/ha
	Planting density：PD	plants/ha
Climate	Average annual temperature：AT	°C
	Average annual precipitation：AP	mm
	Average annual evaporation：AE	mm
Initial soil fertility	Soil organic carbon：SOC	g/kg
	Soil available nitrogen：SAN	mg/kg

Table S2 Sample size and significance test for categorical variables

Categorical variables	Data sample size: cotton yield	Data sample size: water use efficiency	Significance test: cotton yield	Significance test: water use efficiency
Irrigation amount	276	167	0.001	0.001
Drip tape mode	181	114	0.001	0.001
Irrigation frequency	222	133	0.142	0.012
Nitrogen application rate	205	142	0.722	0.005
Variety	215	137	0.001	0.001
Planting density	57	18	0.001	0.982
Annual temperature	108	60	0.001	0.001
Annual evaporation	112	35	0.133	0.207
Annual precipitation	245	163	0.001	0.001
Soil texture	223	157	0.105	0.696
Soil organic carbon	152	93	0.862	0.001
Soil available nitrogen	153	93	0.066	0.503

Table S3 Distribution of titles and locations of literature included in the database

Author	Literature titles	Study site distribution
Shen Xiaojun	Effects of different water treatments on yield and water use efficiency of cotton with drip irrigation under film mulch	The test base of the Irrigation Test Center Station of the Xinjiang Production and Construction Corps
He Pingru	Effects of soil moisture regulation on growth,quality and water use of cotton under drip irrigation in southern Xinjiang	Yuli County, Korla City, Xinjiang
Wang Cheng	Soil salt transfer law and water consumption characteristics for cotton with drip irrigation under mulch with dry sowing and wet seedling	Water-saving Irrigation Experimental Base of School of Water Conservancy and Architectural Engineering, Tarim University, Xinjiang
Yi Pengfei	Schedule optimization of under-plastic-mulch drip irrigation for cotton in arid areas	Key Experimental Station of Agricultural Water-saving Irrigation in Arid Area of Shihezi University
Pan Junjie	Effects of irrigation limits at bud stage and flowering stage on yield of drip irrigation cotton	Modern irrigation demonstration site in Dafeng Town, Hutubi County, Changji Prefecture
WangXiuyuan	Responses of cotton canopy structure characteristics to drip irrigation quota in north XinJiang,China	Shihezi University Experimental Station
Dou Qiaoqiao	Effect of reduced drip irrigation on photosynthetic characteristics and yield of cotton during flowering and bollstagein in Northern Xinjiang	Qubian Village, Shawan County, Xinjiang
Han Xiufeng	Effect of under-mulch-drip irrigation on canopy photosynthesis, canopy structure and yield of hybrid cotton in Xinjiang	Experimental Station of the School of Water Conservancy and Architecture, Tarim University
Wu Fengquan	Effect of plastic film on evapotranspiration and evaporation of cottons under drip irrigation in southern XinJiang	Awati County, Aksu City, Xinjiang Uygur Autonomous Region
Li Nannan	Effects of drip irrigation quota on plant shape,yield and fiber quality of cotton	Agronomy Experimental Station of Shihezi University
Cui Jianping	Influence of different quantities under drip irrigation mode on the long-staple cotton growth and yield	Awati County, Xinjiang
Wang Jichuan	Influence of different drip irrigation amount on canopy structure of hybrid cotton	Experimental Station of the Institute of Water Construction, Tarim University
Wang Jichuan	Effects of irrigation schedule on photosynthetic characteristic and yield components in hybrid cotton Zhaofeng	Experimental Station of the Institute of Water Construction, Tarim University
Gao Shan	Effects of drip irrigation under mulclr on canopy apparent photosynthesis and yield structure of hybrid cotton Zhao feng 1 in south Xinjiang	*
Li Fangsong	Irrigation schedule of drip irrigation of machine-harvested cotton in Hutubi country	Hongliutang Village, Dafeng Town, Hutubi County, Xinjiang

Wang Liang	Effects of irrigation quota on water use efficiency and yield of cotton field under subsoiling tillage	Experimental base of Xinjiang Awati County Academy of Agricultural Sciences
Dou Qiaoqiao	Effect of reduced amount drip irrigation during the flowering and boll-forming on the development of cotton boll and its leaf photosynthetic characteristics	Qubian Village, Sidaohezi Town, Shawan County, Xinjiang
Fan Kai	Experimental research on suitable irrigation management based on meteorological information for film mulched drip irrigation cotton in southern Xinjiang	Soil and Water Conservation Experimental Station of the Water Conservancy Bureau of the First Division of the Xinjiang Production and Construction Corps
Zhao Yongming	Real-time monitor of soil water potential under cotton membrane drip and research on cotton irrigation schedule	Shihezi Institute of Arid Agriculture
Li Nan	Study on the drip irrigation system and the location of soil moisture measuring points under the mulch of mechanized cotton in the south Xinjiang	Irrigation Experimental Station of the Water Conservancy Bureau of the First Division of the Xinjiang Production and Construction Corps
Yang Ying	Effect of water deficit on canopy photosynthetic properties of every green organs in cotton	Agronomy Experimental Station of Shihezi University
He Pingru	Effects of soil water regulation on cotton growth and soil water salt fertilizer transportation under drip irrigation in southern Xinjian	Yuli County, Korla City, Xinjiang
Wang Feng	Effect of irrigation scheduling on growth,yield,and fiber quality of cotton under mechanical harvest cropping model	Aksu National Field Scientific Observation and Research Station of Farmland Ecosystem, Chinese Academy of Sciences
Xing Xiaoning	Effects of different irrigation regimes on cotton growth and yield with drip irrigation under plastic film in oasis region of south Xinjiang	Hydraulic and Architectural Engineering, Tarim University The test base of the hospital
Wu Lifeng	Effect of drip irrigation and fertilizer application on water use efficiency and cotton yield in North of Xinjiang	Water-saving Irrigation Experimental Station of Xinjiang Academy of Agricultural and Reclamation Sciences
Wu Lifeng	Effects of water and fertilizer coupling on cotton yield， net benefits and water use efficiency	Water-saving Irrigation Experimental Station of Xinjiang Academy of Agricultural and Reclamation Sciences
Li Zhijun	Effects of water-fertilizer coupling on field cotton growth and yield under fertigation in Xinjiang	Field Observation and Experimental Station of Crop Efficient Water Use of the Ministry of Agriculture of Shihezi, Xinjiang
Deng Zhong	Effects of water and nitrogen regulation on root and shoot growth characteristics and yield of cotton in arid area	Key Irrigation Experimental Station of Water Conservancy Management Office of Bayingolin Mongolian

		Autonomous Prefecture, Xinjiang
Zhong Zhibo	Effects of different water and nitrogen fertilizers application on growth characteristics and yield of cotton in northern Xinjiang	Shihezi Scientific Observation and Experimental Station of Crop Efficient Water Use of the Ministry of Agriculture
Zhang Yan	Coupling water and fertilizer effects on cotton yield，nitrogen accumulation and nitrogen wse efficiency	Water-saving irrigation experiment of Xinjiang Academy of Agricultural and Reclamation Sciences stand
Liao Huan	Optimal water scheme and N rate for high N uptake and yield of machine-harvested cotton	Shihezi University Agricultural Science Comprehensive Teaching Experimental Center
Yang Shoule	Effects of water and nitrogen coupling on cotton growth characteristics and yield in arid area	Key Irrigation Experimental Station of Water Conservancy Management Office of Bayingolin Mongolian Autonomous Prefecture, Xinjiang
Lin Tao	The effect of deficit irrigation on the yield and water utilization of machine-picked cotton under condition of drip irrigation	Desert Oasis Cultivation and Cultivation Field Comprehensive Observation and Experimental Station of the Ministry of Agriculture and Rural Affairs of Awati County, Xinjiang
Shi Hongliang	Research on the compensation effect of nitrogen fertillizer on cotton yield formation under non-sufficient drip irrigation	Xinjiang Aksu Awati County Harvest 2 Harvest 1 Lian Xinjiang Academy of Agricultural Sciences Economic Workshop
Li Yuyi	Effect of drip irrigation under plastic mulch on aeolian sandy soil salt dynamic and cotton yield	Experimental base of soil improvement station in Shihezi Fort Town, Xinjiang
Xue Fengding	Effects of water-fertilizer coupling on field cotton under fertigation	Shihezi City, Xinjiang Academy of Agricultural Reclamation Sciences Test Site
Wang Haidong	Water-fertilizer coupling effects of field cotton in Xinjiang under fertigation	Shihezi City, Xinjiang Academy of Agricultural Reclamation Sciences Test Site
Zhong Zhibo	Research on irrigation and fertilization system and fertilization strategy of cotton under film	Shihezi Scientific Observation and Experimental Station of Crop Efficient Water Use of the Ministry of Agriculture
Wu Lifeng	Coupling effect of water and fertilizer under fertigation and simulation growth of cotton in Xinjiang	Xinjiang Agricultural Star Academy of Sciences Water-saving Irrigation Experimental Station
Li Xinxin	Water–nitrogen coupling and multi-objective optimization of cotton under mulched drip irrigation in arid northwest China	Shihezi Irrigation District, Xinjiang

Huang Jian	Different sowing dates affected cotton yield and yield components	Shihezi City of the Xinjiang UygurAutonomous Region
Zheng Yaokai	Experimental research on effect of irrigation scheduling on moisture and salt change of mulched drip irrigation for cotton	The 125th Regiment of the Seventh Agricultural Division of the Xinjiang Corps
Huang Zhenzhen	Effect of layout of drip irrigation belt and irrigation quota on soil properties and cotton yield	Cotton Breeding Experimental Base of Xinjiang Agricultural University, Shawan County, Tacheng Prefecture, Xinjiang
Hou Xianghao	Spatial distribution and variability of soil salinity in film-mulched cotton fields under various drip irrigation regimes in southern Xinjiang of China	located in Korla, Xinjiang, China
Zhang Dongmei	Effects of deficit irrigation and plant density on the growth, yield and fiber quality of irrigated cotton	Southern Tumushuke city, Xinjiang, China,
Yang Chuanjie	Effect of deficit irrigation on the growth, water use characteristics and yield of cotton in arid northwest China	Experimental Irrigation Station of Shi-hezi University in Xinjiang, China,
Wang Haidong	Coupling effects of water and fertilizer on yield, water and fertilizer use efficiency of drip-fertigated cotton in northern Xinjiang, China	Test Station of the Xinjiang Academy of Agricultural Reclamation Sciences in Shihezi, Xinjiang Province, China.
He Pingru	Effects of soil water regulation on the cotton yield, fiber quality and soil salt accumulation under mulched drip irrigation in southern Xinjiang, China	Korla city, Xinjiang
Hou Xianghao	Optimal irrigation amount and nitrogen rate improved seed cotton yield while maintaining fiber quality of drip-fertigated cotton in northwest China	Experimental station located in Korla, Xinjiang, China
Li Gan	Membrance under drip irrigation water regulation on machine-harvested cotton photosyntheic characteristic and yield formation mechanism research	The third team of Chuangtian in Liuhudi Town, Manas County, Changji Prefecture, Xinjiang
